# Unsymmetrical Bisacridines’ Interactions with ABC Transporters and Their Cellular Impact on Colon LS 174T and Prostate DU 145 Cancer Cells

**DOI:** 10.3390/molecules29235582

**Published:** 2024-11-26

**Authors:** Monika Pawłowska, Jolanta Kulesza, Ewa Paluszkiewicz, Ewa Augustin, Zofia Mazerska

**Affiliations:** Department of Pharmaceutical Technology and Biochemistry, Faculty of Chemistry, Gdańsk University of Technology, Gabriela Narutowicza Str. 11/12, 80-233 Gdańsk, Poland; monika.pawlowska@pg.edu.pl (M.P.); jolanta.kulesza@pg.edu.pl (J.K.); ewa.paluszkiewicz@pg.edu.pl (E.P.)

**Keywords:** anticancer agents, unsymmetrical bisacridines, ABC transporters, multidrug resistance (MDR), cytotoxicity, cellular response, colon and prostate cancer cell lines

## Abstract

Multidrug resistance (MDR) is a process that constitutes a significant obstacle to effective anticancer therapy. Here, we examined whether unsymmetrical bisacridines (UAs) are substrates for ABC transporters and can influence their expression in human colon LS 174T and prostate DU 145 cancer cells. Moreover, we investigated the cytotoxicity and the cellular response induced by UAs in these cells. The ATPase activities of MDR1, MRP1, and MRP2 were measured using vesicles prepared from insect Sf9 cells expressing particular ABC transporters. The gene expression and protein levels were analyzed using qPCR and Western blotting. The cellular effects were studied by MTT (cytotoxicity), flow cytometry (cell cycle analysis and phosphatidylserine externalization), and fluorescence microscopy. We showed that UAs are substrates for MDR1. Importantly, they did not influence remarkably the expressions of the *ABCB1*, *ABCC1*, and *ABCC2* genes and the levels of the MDR1 and PXR proteins in the studied cells. Furthermore, the cytotoxicity and the level of apoptosis triggered by UAs in LS 174T cells possessing higher expressions of metabolic enzymes were lower compared with DU 145 cells. These results indicate that during possible UA treatment, the occurrence of drug resistance could be limited, which could favor the use of such compounds as potential candidates for future studies.

## 1. Introduction

One of the limitations in successful anticancer therapy outcomes is multidrug resistance (MDR) development. MDR is a phenomenon in which a cancer cell, after acquiring a specific therapy, becomes insusceptible to several other drugs, often differing in both its structure and mode of action. Numerous reasons for MDR development have been presented: reduced drug uptake, drug inactivation, drug target mutations, evading cell death (mainly apoptosis), the self-renewing of cancer resulting from the presence of so-called tumor stem cells, the delineation of features of the tumor microenvironment, including immunosuppression, and the main process: increased drug efflux caused by the upregulation of the expression of ABC transporters that recognize and catalyze the efflux of diverse anticancer drugs from the cells [1,2]. ATP-binding cassette (ABC) transporters are an old superfamily of proteins that participate in the transport of a variety of compounds across the cell membrane and require ATP hydrolysis for their action. In humans, the most important ABC transporters for MDR development are MDR1/P-glycoprotein (*ABCB1*), several members of the MRP (*ABCC*) family (MRP1-9), and the BCRP (*ABCG2*) transporter [3,4]. They are engaged in the efflux of compounds in their native form with a broad substrate selectivity or conjugated with GSH, glucuronic acid, or sulfate [5]. Cancer cells demonstrating MDR generally exhibit ABC transporter overexpression, which is associated with a decreased intracellular drug concentration and, thereby, decreased chemosensitivity [6,7]. MDR-related proteins take part in the efflux of many drugs applied in clinics, like irinotecan, doxorubicin, vinblastine, paclitaxel, mitoxantrone, topotecan, and imatinib. MDR1 was the first discovered and described efflux pump responsible for drug resistance. It is overexpressed in many types of cancers undergoing chemotherapy, like leukemia, breast, ovary, cervical, bladder, lung, and colon cancers, childhood sarcomas, and neuroblastoma [8,9]. Moreover, among ABC transporters, some of the pumps, like MRP2 (*ABCC2*) and MRP3 (*ABCC3*), are known to remove glucuronide conjugates from the cell [10]. Meanwhile, the action of MDR proteins should influence the type and level of cellular response induced in tumor cells [11,12]. Thus, the different levels/expression of ABC transporters in various types of cancer cells result in the changes in therapeutic effectivity observed for anticancer agents [13].

For newly designed compounds, it is necessary to elucidate if they are substrates for ABC transporters and can influence their expression level. Unsymmetrical bisacridines (UAs) are a novel class of anticancer agents that were developed in the Department of Pharmaceutical Technology and Biochemistry of the Gdańsk University of Technology. They were patented in Europe, the USA, Canada, and Japan [14]. These compounds exhibited high cytotoxic activity against fourteen tested cancer cell lines as well as antitumor activity against Walker 256 rat carcinosarcoma and ten human tumor xenografts in nude mice. Among them, there are colon, breast, prostate, lung, and extremely difficult-to-treat pancreatic cancers [15].

**Figure 1:**
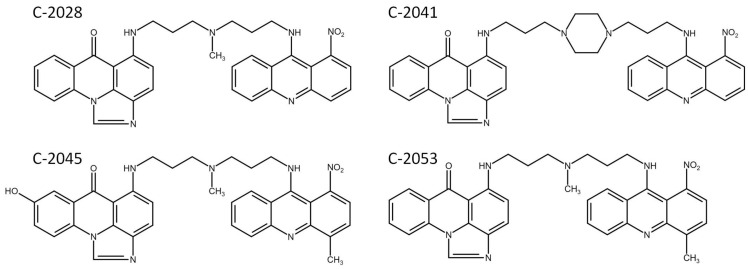
The chemical structures of unsymmetrical bisacridine derivatives: C-2028, C-2041, C-2045, and C-2053 (drawn with ISIS Draw program, version 2.5).

The structures of UAs (Figure 1) contain elements of monomeric compounds previously synthesized in the department: imidazoacridinone, C-1311, and 1-nitroacridine, C-1748, connected by diverse linkers. However, we have shown that UAs’ properties are not a simple sum of the features exhibited by acridine monomers. In this respect, we demonstrated that the general molecular characteristics of UAs, such as their protonation state, self-association ratio, and solubility, are strongly pH-dependent, particularly in the physiological pH range of 6 to 8. Moreover, UAs’ dimers do not intercalate to dsDNA, but they interact with quadruplex DNA (G4) present in specific human guanine-rich sequences with functional significance, such as the ends of chromosomes (telomeres) and the transcriptional regulatory regions of multiple genes, including oncogenes (e.g., the *MYC* and *KRAS* genes) [16].

The molecular mechanism of the biological activity of UAs, as well as their molecular targets, are still being extensively investigated. It has been demonstrated that the strong inhibition of c-Myc by UAs in H460 human non-small cell lung cancer cells could participate in the final cellular response as apoptosis and senescence. Moreover, UAs suppressed cancer spheroid growth, and their anticancer activity was enhanced when bound to quaternary quantum dots, resulting in a selective increase in compound cellular uptake. UAs undergo metabolic transformations, which preserve the compounds’ dimeric structure. Several products of phase I and phase II metabolism have been identified, particularly of uridine UDP-glucuronosyltransferase (UGT)-mediated transformation at the hydroxyl group in the imidazoacridinone ring [17].

Studies on the biological mechanism of antitumor agents should consider the ability of cancer cells to resist the process of multidrug resistance. This is an important impediment to effective therapy. Therefore, we investigated whether the presented bisacridines are substrates for ABC transporters and are able to change membrane pump expression and levels in human cancer cell lines. The involvement of MDR pumps in the transport of compounds was investigated via the measurement of the ATPase activities of MDR1, MRP1, and MRP2 in a non-cellular system using specific vesicles. The changes in the expressions of the *ABCB1*, *ABCC1*, and *ABCC2* genes and the levels of the MDR1 and PXR proteins after UA treatment were evaluated in colon LS 174T and prostate DU 145 cancer cells, which differed in the levels of metabolic enzymes. Finally, we examined whether the interaction of UAs with ABC transporters in the studied cancer cell lines can be reflected in the extent and type of cellular response induced by UAs.

Summing up, the obtained results allow for a better understanding of the mechanism of action of new unsymmetrical bisacridines in tumor cells regarding the role of drug efflux proteins.

## 2. Results

### 2.1. Characteristics of Studied Cell Lines

**Figure 2:**
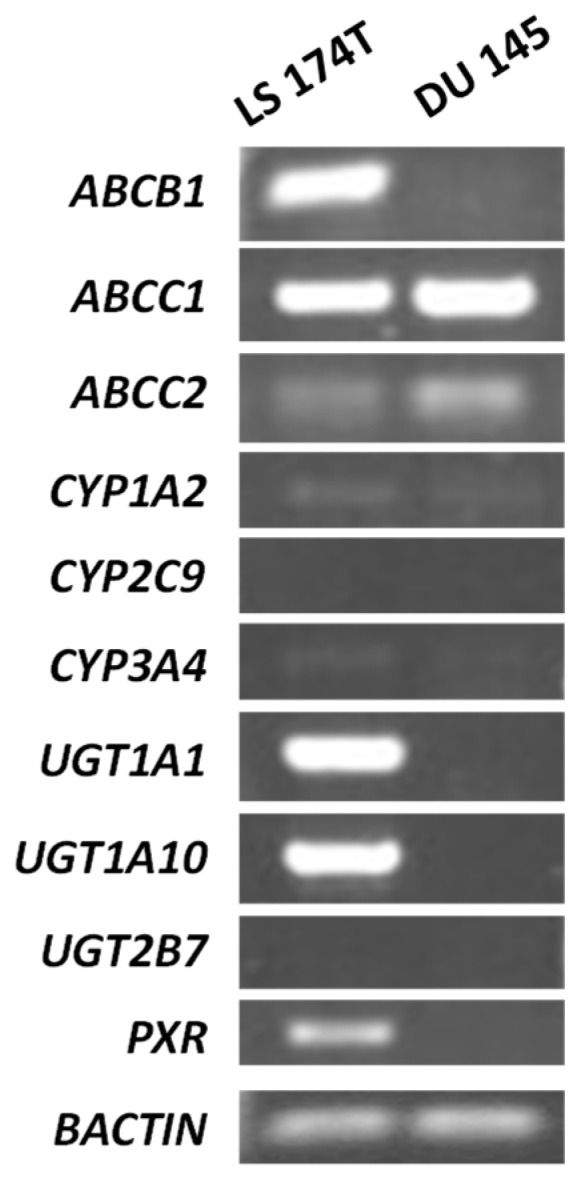
An image of the gel-presenting products of reverse transcription followed by polymerase chain reaction (PCR) analysis of the mRNA expression of the *ABCB1*, *ABCC1*, and *ABCC2* efflux pumps and selected metabolic enzymes, like *CYP1A2*, *CYP2C9*, *CYP3A4*, *UGT1A1*, *UGT1A10*, and *UGT2B7*, and the nuclear receptor PXR in control untreated LS 174T and DU 145 cells. The image of the gel was taken using the UVITEC Cambridge Gel Documentation System.

In the presented studies, we used two varied cell lines: human colon cancer LS 174T (ATCC no. CL-188) and human prostate DU 145 (ATCC no. HTB-81). Both cell lines differ not only in the tumor type origin but also in the metabolic rate and nuclear receptor presence [18,19]. Moreover, both of them possess distinct levels of the tested ABC transporters, allowing us to study the interaction between unsymmetrical bisacridines and efflux pumps depending on the different predispositions of the cells to carry out metabolic transformations [20,21]. Below, in Figure 2, we show an image of the gel-presenting products of the reverse transcription–PCR analysis of the mRNA expression of the ABC transporters and the selected metabolic enzymes, like CYP1A2, CYP2C9, CYP3A4, UGT1A1, UGT1A10, and UGT2B7, which most often participate in the phase I and II biotransformation of xenobiotics, as well as the nuclear receptor PXR, responsible for the regulation of the mentioned proteins.

Reverse transcription followed by PCR revealed that LS 174T and DU 145 cells notably differed in the presence of some of the studied genes, especially those connected to phase II metabolism. The level of the *ABCB1* gene encoding MDR1 was much higher in colon cancer cells than in the prostate cells. Meanwhile, *ABCC1* and *ABCC2* expression was a little bit more pronounced in DU 145 cells. Both cell lines possessed a very low expression of cytochrome P450 genes, including *CYP1A2* and *CYP3A4*; however, LS 174T seemed to exhibit a slightly higher expression of these genes. In the case of the remaining checked genes, LS 174T demonstrated a much greater expression of UGT enzymes, such as the 1A1 and 1A10 isoforms, and the nuclear receptor *PXR*. Both cell lines did not express the *CYP2C9* and *UGT2B7* genes. The colon cancer LS 174T cell line displayed a higher expression of genes pivotal in drug metabolism than DU 145 cells. The levels of some of the ABC transporters were comparable, apart from the MDR1 pump, which differed significantly between colon and prostate cells.

### 2.2. Cytotoxicity of UAs Against LS 174T and DU 145 Cell Lines

The anti-proliferative activity of unsymmetrical bisacridines against LS 174T and DU 145 cells was determined after 72 h of incubation with the tested compounds using an MTT assay. The cells (2000 per well) were seeded on 96-well plates and incubated with increasing concentrations of the UAs up to 10 µM, and concentration-dependent inhibition of cell proliferation was observed. The determined compound concentrations required to inhibit cell growth by 50, 80, and 90% (IC_50_, IC_80,_ and IC_90_ values) are presented in Table 1. The obtained growth inhibition curves are shown in Figures S1 and S2 in the Supplementary Materials.

The determined IC_50_, IC_80_, and IC_90_ values indicated that the LS 174T and DU 145 cells were very sensitive to treatment with the unsymmetrical bisacridines. The UAs inhibited the proliferation of DU 145 cells at lower concentrations than LS 174T cells. For DU 145 cells, the IC_50_ and IC_80_ values for all tested compounds were lower than 0.071 µM. The C-2028 derivative showed the highest activity against cells of both tested lines, with IC_50_ values of 0.018 and 0.007 µM and IC_80_ values of 0.043 and 0.012 µM for LS 174T and DU 145 cells, respectively. The C-2041 compound was slightly less active than C-2028 but more than the C-2053 derivative. The C-2045 bisacridine showed the weakest activity among the tested derivatives, with IC_50_ values that were over five times higher than in the case of C-2028, while the IC_80_ dose was 0.556 µM for LS 174T cells and 0.071 µM for DU 145.

For two compounds, C-2028 and C-2041, we were unable to determine the IC_90_ concentration, as the DU 145 cell line seemed to be more resistant to these compounds than to the other tested compounds (C-2045 and C-2053), and the plotted dose–response curve displayed a plateau effect, suggesting that higher doses did not significantly affect cell viability. Therefore, we decided to use concentrations corresponding to the IC_80_ values for further studies of the UAs’ interactions with the ABC transporters and the cellular response induced by the unsymmetrical bisacridines in the cells of the LS 174T and DU 145 lines.

### 2.3. Interaction of UAs with MDR1, MRP1, and MRP2 Vesicles

It is necessary to find out whether newly designed drugs are substrates for ABC transporters in order to predict the possibility of resistance development. ABC transporters use energy from ATP binding and hydrolysis to transport various substrates across the cell membrane out of the cell. Hence, four unsymmetrical bisacridines, C-2028, C-2041, C-2045, and C-2053, at concentrations of 10 and 100 µM, were incubated with the vesicles (prepared from insect Sf9 cells expressing the particular ABC transporters) containing three different pumps: MDR1, MRP1, and MRP2 (GenoMembrane, Yokohama, Japan). The release of phosphate, as a final product of ATP hydrolysis and the indicator of pump participation in a compound’s removal, was measured. The degree of phosphate formation is presented in Table 2.

The calorimetric measurement of phosphate release (nmol per time of incubation and weight of vesicles) revealed that only MDR1 significantly took part in the transport of the four UA compounds across the membranes at both studied concentrations of derivatives. Using a high dose of the UAs (100 µM), it was shown that the MRP2 pump can also assist in UA compounds’ elimination from the cell.

**Figure 3:**
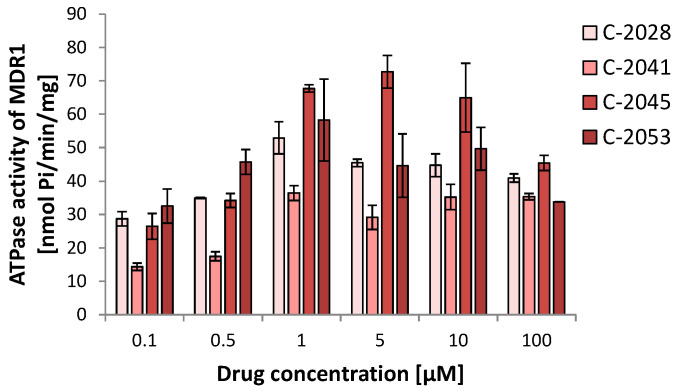
The ATPase activity of the MDR1 pump in the presence of UA compounds. Unsymmetrical bisacridines were incubated for 60 min with 5 ng of vesicles containing the MDR1 transporter (Genomembrane), and the release of phosphate as a product of ATP hydrolysis was measured via the calorimetric method. ATPase activity is described as the quantity of phosphate formed per minute and mg of the vesicles. The bar graphs show the mean activity of the MDR1 pump at the indicated UA concentrations; *n* ≥ 3.

Additionally, the compounds were incubated with vesicles in a wider range of concentrations to predict the efficiency of the MDR1 pump in UAs’ transport, especially at the doses corresponding to the IC_80_ used in the presented studies. The results are shown in Figure 3. At all administrated concentrations of the UAs, the MDR1 protein took part in the transport of the tested compounds, even at very low concentrations, like 0.1 and 0.5 µM. The highest activity of the pump was observed at a UA concentration of 1 µM. The compounds were also efficiently removed at higher doses, even up to 100 µM (not for C-2045, where a small drop was observed). The C-2045 compound presented the highest ratio of transportation by the MDR1 pump, while C-2041 had the lowest.

### 2.4. UAs’ Influence on mRNA Expression of ABC Pumps in LS174T and DU 145 Cells

**Figure 4:**
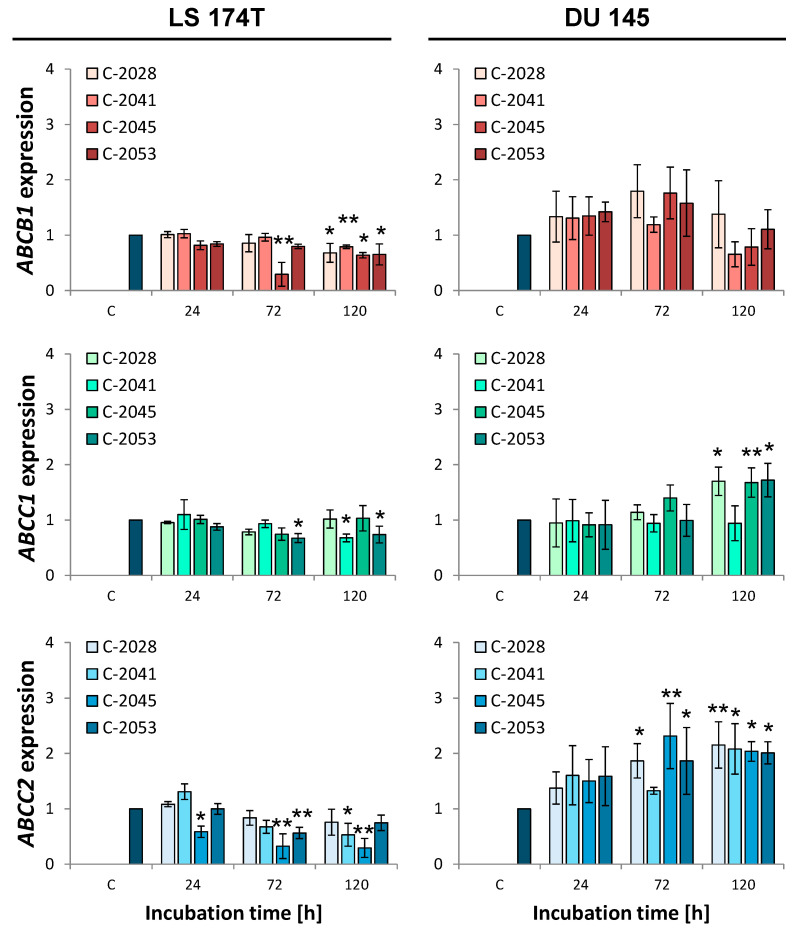
Reverse transcription–quantitative PCR (RT-qPCR) analysis of *ABCB1*, *ABCC1*, and *ABCC2* expression changes in LS 174T and DU 145 cells. The cells were incubated with the IC_80_ doses of the UAs for the times indicated. The total mRNA was isolated and transcribed into cDNA, and real-time quantitative PCR analysis was performed with the appropriate primers for the *ABCB1*, *ABCC1*, and *ABCC2* genes. *GAPDH* was used as a housekeeping gene standard. The relative gene expression was calculated using the 2^−ΔΔCt^ method. The results are presented as means ± SD from at least 3 independent experiments (*n* ≥ 3). The statistics were calculated by an ordinary one-way ANOVA multiple-comparisons test after passing the Shapiro–Wilk normality test using the GraphPad Prism 8.0.2 software. The values were significantly different from the control at * *p* < 0.05 and ** *p* < 0.01.

As the next step in the description of the interaction of the UA compounds with the ABC transporters, we performed reverse transcription–quantitative PCR (RT-qPCR) to depict the influence of the four bisacridine derivatives on the expressions of the *ABCB1*, *ABCC1*, and *ABCC2* genes, encoding the MDR1, MRP1, and MRP2 proteins, respectively, in LS 174T and DU 145 cells. The obtained results are presented in Figure 4.

The evaluation of the expressions of the *ABCB1*, *ABCC1*, and *ABCC2* genes in LS 174T and DU 145 cells revealed that the UA compounds changed the levels of the mentioned genes in the treated cells. The level of the *ABCB1* gene encoding the MDR1 pump significantly decreased with the incubation time with all the tested bisacridines in LS 174T cells, reaching a level of around 0.7 versus the control after 120 h of treatment. In DU 145 cells, the expression level of *ABCB1* slightly increased after 24 and 72 h of incubation with the UAs and then decreased to the initial levels, however, without significance. The expression of the *ABCC1* gene in LS 174T and DU 145 cells changed to some extent upon bisacridine treatment. In the treated C-2041 and C-2053 colon cells, the *ABCC1* gene level slightly decreased, while in prostate cells, it increased after 120 h treatment with C-2028, C-2045, and C-2053. Significant differences in the impact of the UAs on the expression rate of the *ABCC2* gene in colon and prostate cells were observed. In LS 174T cells, the bisacridines, especially the C-2045 compound, led to the downregulation of the gene encoding MRP2. Contrarily, in DU 145 cells, all the UAs caused expression to increase to a level of 2.0 after 120 h.

### 2.5. UAs’ Impact on MDR1 and PXR Levels in DU 145 and LS 174T Cancer Cells

**Figure 5:**
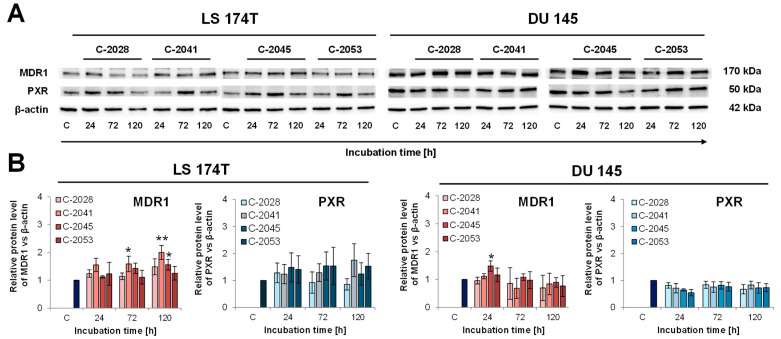
Western blot analysis of MDR1 and PXR protein levels in LS 174T and DU 145 cells. The cells were incubated with the IC_80_ doses of the UAs for the times indicated. Whole-cell extracts were prepared, and 20 μg of proteins/lane was separated by polyacrylamide gel electrophoresis and semi-dry-transferred onto a membrane. The protein levels were detected after immunostaining the membrane with the appropriate antibodies and enhanced chemiluminescence (ECL) development. (**A**) Representative Western blot analysis of MDR1 and PXR in LS 174T and DU 145 cells (the original photos of the Western blots are presented in Figures S3 and S4 in the Supplementary Materials) and (**B**) their relative densitometry quantification performed using Image Lab 6.0.1. The results are presented as means ± SD from at least 3 independent experiments (*n* ≥ 3). The statistics were calculated by an ordinary one-way ANOVA multiple-comparisons test after passing the Shapiro–Wilk normality test using the GraphPad Prism 8.0.2 software. The values were significantly different from the control at * *p* < 0.05 and ** *p* < 0.01.

The MDR1 pump appeared to be responsible for the efflux of the UA compounds in vesicles. Promisingly, the evaluation of the bisacridines’ influence on the expression level of the *ABCB1* gene encoding the MDR1 transporter revealed that its expression was slightly downregulated in LS 174T and not changed in DU 145 cells. Subsequently, we assessed the protein level of MDR1 in colon and prostate cells to be sure that the possibility of resistance development as a consequence of the upregulation of this ABC transporter was limited. Additionally, we checked the protein level of PXR, the nuclear receptor that takes part in MDR1 regulation. Western blot analysis of the MDR1 and PXR protein levels was conducted in LS 174T and DU 145 cells, and the results are presented in Figure 5.

The Western blot analysis showed that remarkable changes in the protein level of MDR1 were not observed in both studied cell lines, LS 174T and DU 145. In LS 174T cells, C-2028 and C-2053 did not alter the transporter level, while C-2041 and C-2045 caused upregulations to the levels of 2.0 and 1.8, respectively, after 120 h of incubation. In DU 145 cells, the bisacridines did not impact the MDR1 protein, which stayed at the same level throughout the whole time of exposure to the UAs (with one exception being C-2045-treated cells only after 24 h). The PXR level was not changed upon UA treatment in both cell lines. In the case of LS 174T, a slight upregulation of the studied nuclear receptor was observed, while in DU 145 cells, downregulation was observed; however, it was without statistical significance.

### 2.6. Cell Cycle Distribution

**Figure 6:**
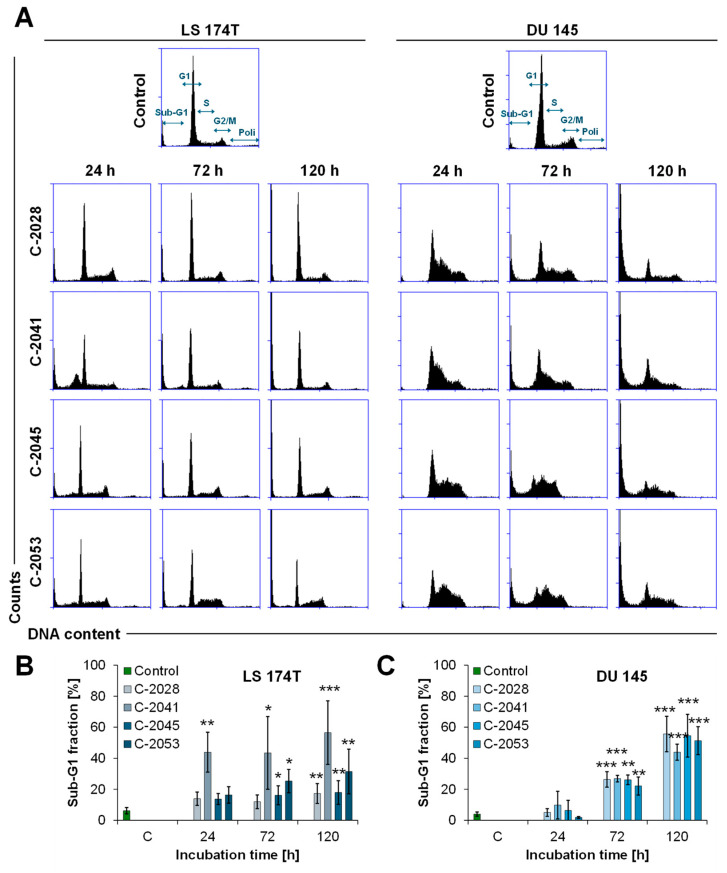
Cell cycle distributions of LS 174T and DU 145 cancer cells. The cells were untreated (control) or treated with the IC_80_ doses of the C-2028, C-2041, C-2045, and C-2053 compounds for the times indicated and subjected to propidium iodide staining and flow cytometry analysis, as described in the Materials and Methods. (**A**) The histograms show the number of cells (*y*-axis) versus the DNA content (*x*-axis) and are representative of at least three experiments for each condition. The bar graphs show the quantified data, expressed as the percentages of LS 174T (**B**) and DU 145 (**C**) cells with less than 2N DNA (sub-G1 fraction). The results are presented as means ± SD from at least 3 independent experiments (*n* ≥ 3). The statistics were calculated by an ordinary one-way ANOVA multiple-comparisons test after passing the Shapiro–Wilk normality test using the GraphPad Prism 8.0.2 software. The values were significantly different from the control at * *p* < 0.05; ** *p* < 0.01; and *** *p* < 0.001.

The analysis of the number of cells in particular phases of the life cycle helped initially determine the effects of the compounds on the cancer cells. LS 174T and DU 145 cells were treated with the IC_80_ dose of the four bisacridine derivatives for 24, 72, and 120 h. The results of the cell cycle analysis of UA-exposed colon and prostate cancer cells are shown in Figure 6, while tables with the detailed values of the percentages of cells in each phase are presented in the Supplementary Materials. The treatment with the UAs caused different responses in each cell line; however, a time-dependent increase in the number of cells with less than 2N DNA was a commonly observed effect.

In LS 174T cells, after 24 h of incubation, the fraction of cells in the G1 phase decreased, while the population of cells in the S and G2/M phases did not differ much from the control and stayed at a similar level throughout the next days of incubation. Significant degradation of the DNA occurred early, after just 24 h of exposure to the UAs, and the sub-G1 cell populations were equal to 13.9, 43.9, 13.7, and 16.4% for C-2028, C-2041, C-2045, and C-2053, respectively. The most profound increase in the sub-G1 fraction was observed for the C-2041 derivative, for which the population of cells with less than 2N DNA was almost 57% after 120 h of incubation. However, this fraction was much lower for other compounds: 31.4% for C-2053 and about 18% for C-2028 and C-2045.

In DU 145 cells treated with the UA compounds, the most characteristic change in the cell cycle distribution was an accumulation of cells in the S phase. After 24 h of exposure, the populations of cells with DNA of 2N-4N corresponded to 38.5, 34, 40.5, and 45% for C-2028, C-2041, C-2045, and C-2053, respectively, and then decreased to around 20% after 120 h of treatment. Concomitant with the increase in cells in the S phase, a drop in the cell populations in the G1 and G2/M phases was observed. Starting from 72 h of treatment with the UAs, the degradation of DNA in DU 145 was notable. After 120 h of exposure, the population of cells with less than 2N DNA was around 51–55.5% for three derivatives: C-2028, C-2045, and C-2053. In DU 145 cells (opposite to LS 174T cells), C-2041 caused the weakest degradation of DNA. Here, after 120 h, the sub-G1 phase corresponded to 43%.

Importantly, the number of the sub-G1 population, considered apoptotic after incubation with the studied compounds, except for C-2041, was notably lower in the case of LS 174T cells compared with DU 145 cells.

### 2.7. Cell Membrane Asymmetry and Integrity Evaluation

**Figure 7:**
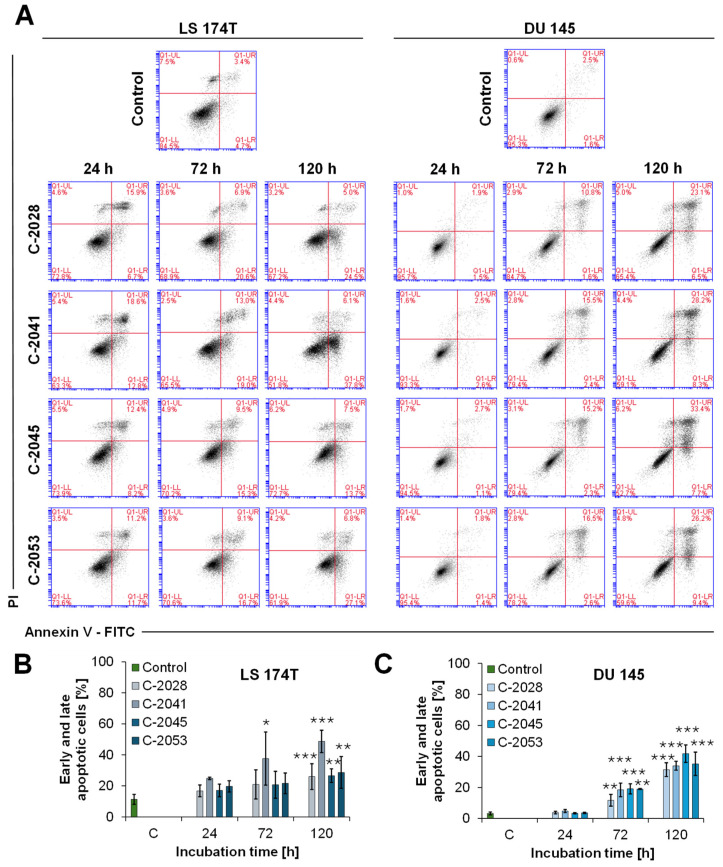
Phosphatidylserine externalization and membrane disruption in LS 174T and DU 145 cancer cells. Cells were untreated (control) or treated with IC_80_ doses of C-2028, C-2041, C-2045, and C-2053 compounds, stained with annexin V–fluorescein isothiocyanate (FITC) and propidium iodide (PI), and flow cytometry analysis was conducted. (**A**) Representative bivariate flow cytometry histograms of annexin V–FITC signals versus PI signals are shown. The bottom left quadrant represents living cells (annexin V-negative; PI-negative); the bottom right quadrant represents early apoptotic cells (annexin V-positive; PI-negative); the top right quadrant represents late apoptotic cells (annexin V-positive; PI-positive); and the top left quadrant represents primary necrotic cells (annexin V-negative; PI-positive). The cytograms shown are representative of at least three independent experiments. (**B**,**C**) The bar graphs show the quantified data, expressed as the percentages of cells stained with annexin V–FITC alone or with PI: the sum of early and late apoptotic cells established for the LS 174T (**B**) and DU 145 (**C**) cell lines. The results are presented as means ± SD from at least 3 independent experiments (*n* ≥ 3). The statistics were calculated by an ordinary one-way ANOVA multiple-comparisons test after passing the Shapiro–Wilk normality test using the GraphPad Prism 8.0.2 software. The values were significantly different from the control at * *p* < 0.05; ** *p* < 0.01; and *** *p* < 0.001.

Along with the changes in the cell cycle distribution, after exposure to the UAs, translocation of phosphatidylserine was observed in both the LS 174T and DU 145 cell lines. This feature is very characteristic of the early stage of apoptosis, where phosphatidylserine is translocated from the inner leaflet of the plasma membrane to the cell surface. Treatment with the studied compounds resulted in a time-dependent increase in annexin V-positive cells, which correlated with the loss of plasma membranes in colon and prostate cells, which can be seen in Figure 7. The detailed values of the percentages of cells in each quadrant are presented in the Supplementary Materials.

The examination of the changes in the cell membrane asymmetry and integrity after UA treatment showed that the altered cellular response (apoptosis) occurred earlier in LS 174T cells compared with DU 145, which was consistent with the cell cycle analysis. In LS 174T cells, about 20% of them were apoptotic just after 24 h of incubation with the UAs. The intensity of the cellular response in colon cancer cells was similar for C-2028, C-2045, and C-2053, and the number of early and late apoptotic cells did not increase much during incubation (to about 25–30% after 120 h). The number of annexin V-positive cells was visibly higher after exposure to C-2041 and reached almost 50% after 120 h.

In DU 145 cells, the majority of cells after 24 h of exposure to the UAs were viable, at about 95%, similar to the control. The most profound membrane alterations in prostate cells were observed after 120 h of treatment with the bisacridines. The C-2045 derivative turned out to be the most effective compound in the induction of apoptosis, and the fraction of annexin V-positive cells after 120 h of incubation reached 41.8%. Early and late apoptotic cells after exposure to C-2028, C-2041, and C-2053 constituted around 30–35%. Meanwhile, the proportion of DU 145 cells undergoing necrosis did not exceed 5%, even after 120 h of incubation with the UAs.

### 2.8. Microscopic Observation of Cell Nuclei

**Figure 8:**
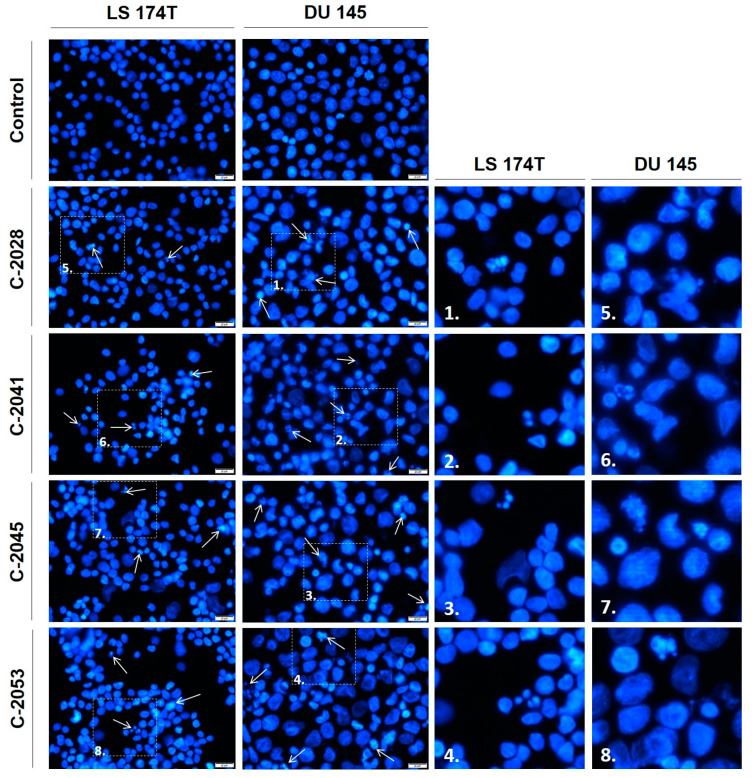
Morphological changes in the nuclei of LS 174T and DU 145 cancer cells treated with the UAs. The pictures (representative of three independent experiments) present the changes in the nuclear morphologies of LS 174T and DU 145 cells treated with the IC_80_ doses of the C-2028, C-2041, C-2045, and C-2053 compounds. The cells were stained with Hoechst 33342 (1 mg/mL) and visualized under a fluorescent microscope (400× magnification). The arrows point at the cells with changes in the nuclear characteristics of apoptosis (condensed, intensely stained, and fragmented chromatin). The white scale bars presented in the images correspond to 20 µm. (**1**–**8**) Enlarged fragments of the pictures with the indicated changes in the characteristics of apoptosis.

Microscopic observations (Figure 8) revealed that exposure to the UAs caused alterations in the cell nuclear morphology in both LS 174T and DU 145 cells. The main observed change was the appearance of features characteristic of apoptosis: chromatin condensation, fragmentation of the cell nucleus, and the presence of apoptotic bodies [22]. In LS 174T cells, some of the cell nuclei presented features typical of apoptosis, and all the tested bisacridines caused similar effects. In DU 145 cells, the strength of induction of apoptosis was comparable for all the UAs; however, it was more pronounced than in colon cancer cells. Moreover, some of the nuclei of both cell lines became slightly enlarged after treatment with the UAs, which was observed mainly after treatment with the C-2045 derivative.

These results confirm that the main type of cellular response induced by the bisacridines in the studied colon and prostate cancer cells was apoptosis, which was induced to a greater extent in DU 145 cells than in LS 174T.

## 3. Discussion

The increased drug efflux caused by the upregulation of ABC transporters is one of the MDR concerns in anticancer therapy [1,2]. Considering the above, we aimed to study the cytotoxic and biological effects of unsymmetrical bisacridines as antitumor agents and to understand their potential to be efflux pump substrates. In this sense, there is the MDR1 protein, the MRP family, and the BCRP protein, which are responsible for xenobiotic removal from tumor cells [5,6,7]. In the beginning, we selected the noncellular model for the transport assessment and performed an analysis of the ATPase activities of the MDR1, MRP1, and MRP2 pumps. Next, we established the influence of UAs on the expression levels of the *ABCB1*, *ABCC1*, and *ABCC2* genes and then the protein levels of MDR1 and the PXR nuclear receptor involved in MDR1’s regulation [18,19]. Finally, we investigated whether the levels of the above-mentioned genes affect the biological response induced by UAs in human cancer cells. We chose the LS 174T and DU 145 cell lines, which differ in the levels of ABC transporters, especially MDR1, and in the metabolic enzymes most often involved in phase I and II xenobiotics’ biotransformation, like CYP1A2, CYP2C9, and CYP3A4 [23], and UGT1A1, UGT1A10, and UGT2B7 [24]. It should be noted that an upregulated level of phase II metabolic enzymes can influence the level of phase III proteins and, thereby, ABC transporters [25].

The results provide us with the initial and crucial finding that UA compounds are substrates for the MDR1 pump (Table 2; Figure 3). Therefore, this ABC transporter should be responsible for drug efflux by a cellular membrane. Table 2 demonstrates that at extremely high concentrations, which usually will not be observed in a living cell (100 µM), the MRP2 pump can also participate in bisacridines’ transport. However, another transporter, MRP1, did not take part in the removal of the studied UA compounds. We also observed differences in the activity of the MDR1 pump in the presence of a particular UA derivative (Figure 3). The ATPase activity of the MDR1 pump was shown to be the highest for C-2045 and the lowest for C-2041 (Figure 3). These compounds are different in the structure of the aminoalkyl link between monomers and in the presence of OH and CH_3_ substituents in the 1-nitroacridine monomer. Thus, C-2041, which differed from the others regarding its lipophilicity, expressed the lowest intensity of transport by the MDR1 pump. Summing up, bisacridines can be added to the list of MDR1 substrates, among other anticancer drugs, such as 5-fluorouracil, actinomycin D, bisantrene, chlorambucil, colchicine, cisplatin, cytarabine, daunorubicin, docetaxel, doxorubicin, epirubicin, etoposide, gefitinib, hydroxyurea, irinotecan (CPT-11), methotrexate, mitomycin C, mitoxantrone, paclitaxel, tamoxifen, teniposide, topotecan, vinblastine, and vincristine [26].

Human colon LS 174T and human prostate DU 145 cells were selected as the in vitro model for the following investigations. As mentioned earlier, this selection resulted from differences in the level of the *ABCB1* gene and those that encode metabolic enzymes, particularly responsible for phase II metabolic detoxifications (Figure 2). In LS 174T cells, elevated levels of the *ABCB1*, *UGT1A1*, *UGT1A10*, and *PXR* genes were observed in comparison with those in DU 145 cells. What is most important is that UA treatment in LS 174T cells influenced the expressions of the *ABCB1*, *ABCC1*, and *ABCC2* genes to some extent in comparison with control cells, leading to a decrease in their expression (Figure 4 of qPCR). In contrast, the action of the UAs toward *ABCC1* and *ABCC2*’s expression in DU 145 cells was increased, particularly after 120 h of incubation, whereas *ABCB1*’s expression essentially did not change (Figure 4). To improve the above results, Western blotting analysis was undertaken, which showed that the MDR1 protein levels increased after treatment with C-2041 and slightly with C-2045 in LS 174T cells (Figure 5). In other cases, the level of the MDR1 protein was not changed notably. This shows that the UA compounds, especially C-2028, C-2045, and C-2053, did not remarkably change the level of the MDR1 pump, which participated in their efflux.

Moreover, the PXR protein, the nuclear receptor participating in the regulation level of MDR1 and responsible for the induction of metabolic/detoxification enzymes, was not significantly changed in this respect. Thus, the level of detoxification metabolism will not be changed after UA treatment and will not influence the drug resistance of these compounds. This observation is promising because compounds that do not impact the level of PXR and its downstream proteins might limit potential side effects [27].

The results presented are desirable because during therapy with the UAs, the possible resistance development can be limited, and, generally, the intracellular concentration of the bisacridines should stay at the proper level, except for C-2041 (Figure 4 and Figure 5). Usually, the level of the pump participating in the removal of a particular drug is significantly elevated after its exposure, which was presented for imatinib removed by MDR1 [6] or doxorubicin transported by MDR1 and MRP1 [28]. Interestingly, C-2041, which showed the lowest possibility of being removed from the cell (Figure 3), causing the lowest activity of the MDR1 pump, led to an increase in MDR1 levels in LS 174T cells.

These results are consistent with the cytotoxicity of the bisacridines toward the studied colon and prostate cancer cells. The results presented in Table 1 demonstrate that LS 174T colon cells showing a higher expression of *ABCB1* and metabolic enzymes were generally less sensitive to the studied compounds in comparison with prostate DU 145 cells. Considering a potential structure–activity relationship, C-2045, the only one with a hydroxyl group in the imidazoacridinone ring together with a methyl substituent in the 1-nitroacridine moiety, expressed the lowest activity against both cell lines, while LS 174T cells turned out to be less sensitive to this derivative than DU 145. We suggest that the structure of C-2045, which differs from the other UAs in the presence of a hydroxyl group, is responsible for its specific action. The hydroxyl group is susceptible to metabolic glucuronidation, which usually decreases the activity of the compound and makes drug efflux easier, which shows ATPase activity (Figure 3) [25,29].

The main cellular response triggered by the UA compounds in both LS 174T and DU 145 cells was apoptosis. The induction of this type of cell death was confirmed by cell cycle analysis (Figure 6), phosphatidylserine externalization, and nuclear morphological observation (Figure 7 and Figure 8). LS 174T colon cancer cells appeared to be less sensitive to the UAs’ action than DU 145 prostate cancer cells. With respect to cell death, the other results are in agreement with the above, indicating that colon cells are usually more often exposed to toxic compounds, which makes them less susceptible to detrimental effects [30]. Therefore, the induction of apoptosis referred to a smaller population of LS 174T cells than in the case of DU 145.

A specific exception to the results presented above can be observed for compound C-2041, characterized by a structure standing out from the others (Figure 1). It possesses a specific aminoalkyl chain linking with the cyclic aminoalkyl element, and there is no substituent present in the aromatic ring. Some results presented previously revealed the non-selective action of C-2041 toward some types of tumor cells, particularly toward HCT 116 and, likewise, colon cancer cells [17]. In the present work, we found that C-2041 induced the lowest ATPase activity in the non-cellular system (Figure 3) and was the only one that strongly modified the level of the MDR1 protein in LS 174T cells (Figure 5). Moreover, it induced a significant and outstanding, compared with the other compounds, increase in the level of cell death in LS 174T tumor cells (Figure 6 and Figure 7). Thus, we suggest that the high level of the sub-G1 population as a marker of apoptotic cell death observed for C-2041 resulted from the decrease in ATPase activity, leading to the inhibition of the compound’s efflux and subsequently causing the upregulation of the MDR1 protein. As a result, a slight increase in drug resistance would be observed for C-2041, like for other clinically used compounds [31,32,33].

Summing up, we found that unsymmetrical bisacridines are substrates for the MDR1 pump. We suspect that ABC transporters should be responsible for their efflux under physiological conditions. What is most important is that these compounds only slightly influenced the expression of the *ABCB1*, *ABCC1*, and *ABCC2* genes and the level of the MDR1 protein in the studied colon LS 174T and prostate DU 145 tumor cells, especially for the C-2028, C-2045, and C-2053 derivatives. Such a result is desirable and promising because it indicates that during possible treatment, the development of compound resistance can be limited. Thus, we found that antitumor unsymmetrical bisacridines might be able to avoid high levels of drug resistance.

## 4. Materials and Methods

### 4.1. Chemicals and Reagents

Unsymmetrical bisacridine derivatives (UAs), C-2028, C-2041, C-2045, and C-2053, were synthesized in the Department of Pharmaceutical Technology and Biochemistry, the Gdańsk University of Technology, according to a previously published procedure [15]. All compounds were prepared as monochlorides to achieve the best solubility and stability. Stock and working solutions of all compounds were prepared in sterile and deionized water (Milli-Q, Merck, Darmstadt, Germany).

Vesicles containing the MDR1, MRP1, and MRP2 pumps were obtained from GenoMembrane (Yokohama, Japan). Anti-MDR1, anti-PXR, and anti-β-actin antibodies were ordered from Sigma-Aldrich (Merck, St. Louise, CA, USA). Secondary anti-mouse and anti-rabbit antibodies linked to horseradish peroxidase were purchased from Cell Signaling Technology (Beverly, MA, USA). PI/RNase Staining Buffer and the FITC Annexin V Apoptosis Detection Kit were obtained from BD Biosciences (San Diego, CA, USA). Fetal bovine serum (FBS) was purchased from Biowest (Nuaille, France). RIPA buffers were purchased from Abcam (Cambridge, Great Britain). Maxima Hot Start Green PCR Master Mix, GeneRuler 50 bp DNA Ladder, Enhanced Chemiluminescence System Reagents, and Restore PLUS Western Blot Stripping Buffer were obtained from Thermo Scientific (Waltham, MA, USA). The High Pure RNA Isolation Kit, Transcriptor First Strand cDNA Synthesis Kit, LightCycler^®^ 480 SYBR Green I Master, Phosphatase Inhibitor Cocktail Tablets PhosSTOPTM, and Mini EDTA-free Protease Inhibitor Cocktail cOmpleteTM were purchased from Roche (Manheim, Germany). The primers for the PCR reaction were ordered from Genomed (Warsaw, Poland). Laemmli buffer, nitrocellulose membranes, EveryBlot Blocking Buffer, and the DC Protein Assay were obtained from Bio-Rad (Hercules, CA, USA). The following reagents were purchased from Merck (Sigma-Aldrich; Darmstadt, Germany): 3-(4,5-dimethylthiazol-2-yl)-2,5-diphenyltetrazolium bromide (MTT), acrylamide/bis-acrylamide 30% solution, agarose, ammonium persulfate, ascorbic acid, disodium adenosine 5′-triphosphate (ATP.2Na), dithiothreitol (DTT), ethylene glycol-bis (β-aminoethyl ether)-N,N,N′,N′-tetraacetic acid tetrasodium (EGTA), EMEM medium, ethidium bromide, ethylenediaminetetraacetic acid (EDTA), glycine, hexaammonium heptamolybdate tetrahydrate, Hoechst 33342, magnesium chloride, MOPS buffer, N,N,N′,N′-tetramethylethylenediamine (TEMED), penicillin–streptomycin solution, phenylmethanesulfonyl fluoride (PMSF), phosphate-buffered saline (PBS), potassium chloride, sodium azide (NaN_3_), sodium dihydrogen phosphate, sodium deoxycholate, sodium dodecyl sulfate (SDS), sodium hydroxide, sodium orthovanadate, Tris base, Tris hydrochloride, trypsin–EDTA solution, Tween 20, and zinc acetate dihydrate. The following reagents were purchased from POCH S.A. (Gliwice, Poland): dimethyl sulfoxide, ethanol, methanol, sodium chloride, sodium fluoride, and acetic acid. Ultra-pure deionized water R >18 MΩ cm^−1^ was prepared by the Milli-Q Integral Water Purification System, Merck Millipore (Billerica, MA, USA).

### 4.2. Cell Lines and Culture

In the presented work, two cell lines were used: human colon cancer, LS 174T (ATCC no. CL-188), and human prostate cancer, DU 145 (ATCC no. HTB-81). Both cell lines were purchased from the American Type Culture Collection (Manassas, VA, USA, ATCC) and were tested negatively for mycoplasma using the Universal Mycoplasma Detection Kit (ATCC). The LS 174T and DU 145 cells were maintained in Eagle’s minimal essential medium (EMEM; Merck, Darmstadt, Germany) and supplemented with 10% fetal bovine serum (FBS; Biowest, Riverside, MO, USA), 100 μg/mL of streptomycin, and 100 U/mL of penicillin (Merck, Darmstadt, Germany). The cells were incubated in a 5% CO_2_ atmosphere at 37 °C. The experiments were performed with cells in the exponential phase of growth.

### 4.3. mRNA Isolation and Reverse Transcription–Polymerase Chain Reaction

The relative expressions of *ABCB1*, *ABCC1*, *ABCC2*, *CYP1A2*, *CYP2C9*, *CYP3A4*, *UGT1A1*, *UGT1A10*, *UGT2B7*, and *PXR* in LS 174T and DU 145 cells were determined by reverse transcription–polymerase chain reaction. mRNA was isolated from 1 × 10^6^ confluent untreated cells using the High Pure RNA Isolation Kit according to the manufacturer’s instructions (Roche, Basel, Switzerland). The concentration of RNA was determined by a NanoDrop 2000 (Thermo Scientific, Pittsburgh, PA, USA). Then, 1 μg of RNA was reverse-transcribed using the Transcriptor First Strand cDNA Synthesis Kit according to the manufacturer’s instructions (Roche Diagnostics, Mannheim, Germany) in 20 μL of the reaction mixture. The reverse transcription reaction was carried out for 30 min at 55 °C and stopped by heating to 85 °C for 5 min and placing on ice. The cDNA samples achieved from both studied cell lines were subjected to PCR reaction in a thermal cycler (Thermo Scientific, Eppendorf, Pittsburgh, PA, USA) with the following conditions: 1 cycle for 4 min at 95 °C (preincubation); 45 cycles of amplification for 30 s at 95 °C (denaturation), 30 s at 61 °C (annealing), and 30 s at 72 °C (extension); 1 cycle for 5 min at 72 °C (final extension); and 1 cycle for 30 s at 4 °C (cooling). The primers were ordered from Genomed (Warsaw, Poland), and the sequences were as follows: *ABCB1* forward primer—CCCATCATTGCAATAGCAGG; *ABCB1* reverse primer—TGTTCAAACTTCTGCTCCTGA [34]; *ABCC1* forward primer—CGTGTGGGTGCCTTGTTTTT; *ABCC1* reverse primer—GCACACACTAGGGCTACCAG; *ABCC2* forward primer—TCGAACACTTAGCCGCAGTT; *ABCC2* reverse primer—TCTTCGTCTTCCTTCAGGCT; *CYP1A2* forward primer—TCAGCCTCGTGAAGAACACTC; *CYP1A2* reverse primer—GACTGTGTCAAATCCTGCTCC; *CYP2C9* forward primer—AGAACCTTGACACCACTCCAG; *CYP2C9* reverse primer—AGGCATTACAGATAGTGAAAGATGG; *CYP3A4* forward primer—TTTCCACCACCCCCAGTTAG; *CYP3A4* reverse primer—CCACGCCAACAGTGATTACA; *UGT1A1* forward primer—CCTTGCCTCAGAATTCCTTC; *UGT1A1* reverse primer—ATTGATCCCAAAGAGAAAACCAC; *UGT1A10* forward primer—CCTCTTTCCTATGTCCCCAATGA; *UGT1A10* reverse primer—CCTTAGTCTCCATGCGCTT TGC [35]; *UGT2B7* forward primer—AGTTGGAGAATTTCATCATGCAACAGA; and *UGT2B7* reverse primer—TCAGCCCAGCAGCTCACCACAGGG. The primers were designed using the online tool Primer-BLAST (NCBI, Bethesda, MD, USA) or according to the literature, if stated. Equal amounts of aliquots of the PCR products were separated via electrophoresis (90 V; 30 min) on 1.8% agarose gel with a Mini-Sub Cell GT (Bio-Rad Laboratories, Hercules, CA, USA) and visualized by ethidium bromide staining. Photos of the gels were taken by the UVITEC Cambridge Gel Documentation System (UVITEC Cambridge, Cambridge, Great Britain).

### 4.4. Measurement of ATPase Activity of Selected ABC Transporters

The ATPase activity of the MDR1, MRP1, and MRP2 pumps was measured using specific vesicles expressing the mentioned ABC transporters (GenoMembrane, Yokohama, Japan) according to the manufacturing protocol of ATPase assay (GenoMembrane, Yokohama, Japan). Briefly, solutions in a reaction buffer consisting of standard phosphate dilutions (to obtain a calibration curve), orthovanadate, MgATP, the test compounds, a stop solution (10 *w/v*% SDS), and 2 mg/mL of ABC transporter vesicles (with a final protein amount of 0.02 mg) were prepared. Before each assay’s performance, detection reagent solution 1 (containing acetic acid) and detection reagent solution 2 (containing hexaammonium heptamolybdate tetrahydrate and zinc acetate dihydrate) were formulated. First, the standard phosphate dilutions, ABC transporter vesicle solutions, test compounds (the UA derivatives at 3-fold concentrations (final from 0.01 to 100 µM)), positive controls at 3-fold concentrations (50 µM verapamil, 10 mM NEM-GS, and 1 mM probenecid for the MDR1, MRP1, and MRP2 vesicles, respectively), stop solution, reaction buffer, and orthovanadate solution were placed on a 96-well microplate according to the protocol plate layout, gently mixed, and incubated at 37 °C for 3 min. Then, a 12 mM MgATP solution was added to the proper wells, and the plate was incubated at 37 °C for the appropriate time (60 min for the UAs and positive controls). The reaction was stopped by adding 30 µL of the stop solution. An amount of 20 mL of detection reagent solution 1 and 5 mL of detection reagent solution 2 were mixed, and 200 µL of the mixed solution was added to all wells. The plate was incubated at 37 °C for 20 min to allow for the color reaction to occur, and the absorbance was measured at 780 nm. The sensitive ATPase activity based on the amount of generated inorganic phosphate was calculated as follows:

sensitive ATPase activity (nmol Pi/min/mg protein) = [generated inorganic phosphate (nmol)] ÷ [reaction time (min)] ÷ [protein amount (mg)]

### 4.5. Cell Growth Inhibition Assay

To investigate the effect of UAs on cell viability, a colorimetric analysis was performed using MTT (3-(4,5-dimethyl-2-thiazolyl)-2,5-diphenyl-2H-tetrazolium bromide. LS 174T and DU 145 cells (2000/well) were seeded in 96-well microplates, and the following UAs were added at concentrations of up to 10 μM: C-2028, C-2041, C-2045, and C-2053. Stock solutions (10 mM) were prepared in sterile water, as well as dilutions. After 72 h, 20 µL of MTT solution at a concentration of 4 mg/mL (800 μg/well) was added for 3 h, and then the formazan crystals were dissolved in DMSO, and the absorbance was measured at 540 nm using an iMark Microplate Absorbance Reader (Bio-Rad, Hercules, CA, USA). The compound concentrations required to inhibit cell growth by 50 (IC_50_) and 80% (IC_80_) compared with untreated control cells were determined from curves plotting survival as a function of dose. The calculations were performed according to the following equation:

z = (A_1_ × 100%)/A_2_

where A_1_ is the mean absorbance for cells treated with the compound [-], and A_2_ is the mean absorbance for control cells [-].

Next, a graph of the dependence of cell growth inhibition on the logarithm of the concentration of the tested substance was drawn. A rectilinear fragment and a logarithmic equation of the trend line were determined, which had the general form:

y=a ln(x)+b

where y is the dependent variable. In the case of the IC_50_ parameter, which means the concentration of the compound at which cell proliferation in an in vitro culture is inhibited by 50% compared with control cells, it is 50, whereas, for the IC_80_, which means the concentration of the compound needed to inhibit 80% of the cells, it is 20. a and b are constants, and x is the concentration of the tested compound [µM].

In order to determine the IC_50_ and IC_80_, the logarithmic equation was transformed into the form $x=e^{\frac{y-b}{a}}$, the specific data were substituted, and the desired parameters were determined. Results were obtained from at least six independent experiments with four replicates for each concentration.

### 4.6. Reverse Transcription–Quantitative Polymerase Chain Reaction (RT-qPCR)

The procedures for mRNA isolation from LS 174T and DU 145 cells and their subsequent reverse transcription to cDNA using reverse DNA polymerase are presented in Item 4.3. of the Materials and Methods Section. Reverse transcription–quantitative PCT (RT-qPCR) was performed on 2 µL of cDNA using 0.2 µM of the forward and reverse primers on LightCycler^®^ 480 using the LightCycler^®^ 480 SYBR Green I Master according to the manufacturer’s instructions (Roche). The primers for the *ABCB1*, *ABCC1*, and *ABCC2* genes are presented in Item 4.3. of the Materials and Methods Section. The reference genes (*GAPDH* and *BACTIN* for LS 174T and DU 145 cells, respectively) were designed using the online tool Primer BLAST (NCBI, Rothesde, MD, USA), and their sequences were as follows: *GAPDH* forward primer—ACCCACTCCTCCACCTTTG; *GAPDH* reverse primer—CTCTTGTGCTCTTGCTGGG; *BACTIN* forward primer—CTTCGCGGGCGACGAT; and *BACTIN* reverse primer—CCACATAGGAATCCTTCTGACC. The cycling conditions were as follows: 1 cycle for 7 min at 95 °C (preincubation); 45 cycles of amplification for 10 s at 95 °C (denaturation), 20 s at 62 °C (annealing), and 30 s at 72 °C (extension); 1 cycle for obtaining the melting curve; and 1 cycle for 10 s at 40 °C (cooling). The results were standardized to the reference genes *GADPH* and *BACTIN* for LS174T and DU 145 cells, respectively. The relative expression levels of the *ABCB1*, *ABCC1*, and *ABCC2* genes were quantified using the comparative method 2^ΔΔCt^ [36]. The results were obtained from three independent experiments, each of which was analyzed at least twice.

### 4.7. Western Blotting

LS 174T and DU 145 cells were seeded and treated with the UA compounds at the IC80 doses. After exposure to the compounds, the cells were scraped, pooled with floating cells, and washed twice with ice-cold PBS. The cells were suspended in RIPA buffer (Abcam, Cambridge, Great Britain) with a cocktail of protease and phosphatase inhibitors (Roche) and 1 mM phenylmethanesulfonyl fluoride (PMSF) and maintained for 20 min on ice with brief vortexing every 5 min. The lysates were then centrifuged at 14,000× *g* for 15 min at 4 °C. The protein concentration was determined using the DC Protein Assay (Bio-Rad, Hercules, CA, USA). The samples were mixed with Laemmli buffer (Bio-Rad) and denatured at 100 °C for 5 min. Then, 30 μg of total protein was subjected to SDS-PAGE and transferred onto nitrocellulose membranes using a semi-dry blotting apparatus (Bio-Rad, Hercules, CA, USA). The membrane was blocked with EveryBlot Blocking Buffer Bio-Rad, Hercules, CA, USA), washed five times with TBST buffer, and probed with primary antibodies. Mouse anti-MDR1, rabbit anti-PXR, and mouse anti-β-actin antibodies were ordered from Sigma-Aldrich (St. Louis, CA, USA). Secondary anti-mouse and anti-rabbit horseradish peroxidase-linked antibody was purchased from Cell Signaling Technology (Danvers, MA, USA). If necessary, to re-probe the blot with another antibody, the membrane was washed 5 times with TBST after developing the first antibody. The membrane was then treated with Restore PLUS Western Blot Stripping Buffer (Thermo Scientific, Waltham, MA, USA) for 15 min with gentle shaking. Next, the membrane was washed with TBST, again blocked with the blocking buffer, washed, and probed with another primary antibody, as described above. For each Western blot, chemiluminescence detection was performed using Enhanced Chemiluminescence System Reagents (Thermo Scientific, Waltham, MA, USA). Densitometric analysis was performed using the Image Lab software 6.0.1 (Bio-Rad, Hercules, CA, USA). The experiment was performed at least three times.

### 4.8. Cell Cycle Analysis

The cell cycle distribution was assessed by the DNA content, which was determined by flow cytometry using propidium iodide (PI), a fluorescent dye that binds stoichiometrically to DNA. LS 174T and DU 145 cells were treated with the UAs at the IC_80_ doses for 24 to 120 h. Following exposure, 2.0 × 10^6^ cells were trypsinized, pooled with floating cells, washed twice with cold PBS to minimize cell activity and the possibility of cell death occurrence during cell preparation, and fixed in 80% (*v*/*v*) ethanol at −20 °C for at least 24 h. Afterward, the fixed cells were washed twice with PBS, stained with PI/RNase Staining Buffer (BD Biosciences, San Jose, CA, USA), and analyzed using a FACS Accuri C6 flow cytometer (BD Biosciences, San Jose, CA, USA). At least 10,000 cells were collected, and the results were analyzed using the BD Accuri C6 Software (Version 1.0264.21). The experiment was performed at least three times.

### 4.9. Annexin V/Propidium Iodide (PI) Analysis

The type of death induced by the test compounds was assessed using double staining with the Annexin-V Fluos Staining Kit (BD Biosciences, San Jose, CA, USA), allowing the identification of cells with the externalization of phosphatidylserine (a hallmark of apoptosis) and dead cells according to the manufacturer’s protocol. Briefly, after compound treatment, the LS 174T and DU 145 cells (1.5 × 10^6^) were trypsinized, washed twice with cold PBS, pelleted, and resuspended in 50 µL of a binding buffer containing Annexin V–FITC and PI. The cells were incubated in the dark for 15 min at room temperature and then diluted with 180 µL of the binding buffer and analyzed by flow cytometry with FACS Accuri C6 (BD, San Jose, CA, USA). At least 10,000 cells were collected, and the results were analyzed using the BD Accuri C6 software (version 1.0264.21). The experiment was performed at least four times. Cells with low FITC and PI fluorescence were considered viable. Cells that presented high FITC fluorescence but low PI fluorescence were regarded as early apoptotic. Late apoptotic cells presented high FITC and PI fluorescence. Necrotic cells had low FITC fluorescence and high PI fluorescence.

### 4.10. Assessment of Cell Nuclear Morphology

Nuclear morphology was examined under a fluorescence microscope after staining with a fluorescence dye, Hoechst 33342. Briefly, after compound treatment (IC_80_ dose), LS 174T and DU 145 cells were spun onto microscopic slides, fixed with methanol–acetic acid (3:1) for 15 min, and stained with Hoechst 33342 (1 mg/mL) for 15 min. The morphology of the nuclei was examined under an OLYMPUS BX60 microscope connected to a U-RFL-T lamp coupled via an XC50 CCD camera to a personal computer equipped with the cellSens Standard 1.18 software (Olympus, Tokyo, Japan). Pictures were taken using the UV filter and 400× magnification. Cells were identified as apoptotic based on the presence of condensed, fragmented chromatin. The experiment was performed at least three times.

### 4.11. Data Analysis

All data are presented as the means of the results from at least 3 independent experiments ± standard deviation. The normality of the data was assessed using the Shapiro–Wilk test. Statistical analysis was performed using the ordinary one-way ANOVA multiple-comparisons test of variance for parametric data. Differences of *p* < 0.05 between the group of untreated cells (negative control) and the group of cells treated with the compounds were considered statistically significant according to the following criteria: * *p* < 0.05, ** *p* < 0.01, and *** *p* < 0.001. Statistical analysis of the data was performed using GraphPad Prism 8.0.2 (GraphPad Software, San Diego, CA, USA).

## 5. Conclusions

The aim of the presented study was to explain the role of drug efflux in the cytotoxic and biological effects of unsymmetrical bisacridine antitumor agents. It resulted from the observed limitations in successful anticancer therapy induced by the upregulation of ABC transporters responsible for the compounds’ removal from the cell.

It was found that unsymmetrical bisacridines were substrates for the MDR1 pump. Thus, we suspected that this ABC transporter should be responsible for UAs’ efflux under physiological conditions. What is most important is that these compounds only slightly influenced the expressions of the *ABCB1*, *ABCC1*, and *ABCC2* genes. Moreover, the level of the MDR1 protein in the investigated colon LS 174T and prostate DU 145 cells was only slightly changed. The main observed cellular effect induced by the UA compounds in LS 174T and DU 145 cells was apoptosis, the extent of which may be increased in the case of limited drug efflux, which was shown in the case of the C-2041 derivative.

The results obtained are desirable and promising because they indicate that during possible treatment, the development of UA resistance can be limited, which could favor the use of such compounds as potential candidates for future studies.

## Figures and Tables

**Table 1:** Cytotoxicity of UAs against LS 174T and DU 145 cells.

Cell Line	Compound	IC_50_ [µM]	IC_80_ [µM]	IC_90_ [µM]
LS 174T	C-2028	0.018 ± 0.007	0.043 ± 0.012	0.060 ± 0.015
	C-2041	0.051 ± 0.010	0.113 ± 0.030	0.288 ± 0.047
	C-2045	0.126 ± 0.033	0.556 ± 0.119	1.333 ± 0.753
	C-2053	0.050 ± 0.011	0.161 ± 0.022	0.358 ± 0.042
DU 145	C-2028	0.007 ± 0.001	0.012 ± 0.003	-
	C-2041	0.013 ± 0.003	0.032 ± 0.007	-
	C-2045	0.033 ± 0.008	0.071 ± 0.017	0.132 ± 0.099
	C-2053	0.022 ± 0.005	0.045 ± 0.007	0.061 ± 0.026

IC_50_, IC_80_, and IC_90_ values—compound concentrations required to inhibit cell growth by 50, 80, and 90% versus control untreated cells. Means from at least 6 independent assays from at least 4 replicates for each compound’s concentrations.

**Table 2:** Activities of MDR1, MRP1, and MRP2 pumps in the presence of UAs.

Compound	ATPase Activity [nmol/min/mg]		
	MDR1	MRP1	MRP2
	10 µM of UAs		
C-2028	44.7 ± 3.4	0	0
C-2041	35.2 ± 3.8	0	0
C-2045	64.9 ± 10.3	0	0
C-2053	49.7 ± 6.4	0	0
	100 µM of UAs		
C-2028	40.9 ± 1.2	0	8.6 ± 1.4
C-2041	35.4 ± 0.9	0	4.7 ± 4.9
C-2045	45.4 ± 2.3	0	2.4 ± 0.6
C-2053	33.8 ± 0.1	0	6.7 ± 4.1

## Data Availability

All data generated or analyzed during this study are included in this published article and the Supplementary Materials Files.

## Supplementary Materials

The following supporting information can be downloaded at https://www.mdpi.com/article/10.3390/molecules29235582/s1. Figure S1: Growth inhibition curves of LS 174T cells after exposure to UA compounds; Figure S2: Growth inhibition curves of DU 145 cells after exposure to UA compounds; Figure S3: Original photos taken for reverse transcription–PCR analysis, which are presented in Figure 2; Figure S4: Original photos taken for Western blotting analysis, which are presented in Figure 5, taken for LS 174T cells; Figure S5: Original photos taken for Western blotting analysis, which are presented in Figure 5, taken for DU 145 cells; Table S1: Cell cycle distribution of LS 174T cells after treatment with C-2028, C-2041, C-2045, and C-2053 (at IC_80_ dose); *n* ≥ 3; Table S2: Cell cycle distribution of DU 145 cells after treatment with C-2028, C-2041, C-2045, and C-2053 (at IC_80_ dose); *n* ≥ 3; Table S3: Analysis of asymmetry and permeability of cell membrane in LS 174T cells after treatment with C-2028, C-2041, C-2045, and C-2053 (at IC_80_ dose); *n* ≥ 4; Table S4: Analysis of asymmetry and permeability of cell membrane in DU 145 cells after treatment with C-2028, C-2041, C-2045, and C-2053 (at IC_80_ dose); *n* ≥ 4.
