# Supplementary material for: Unsymmetrical Bisacridines’ Interactions with ABC Transporters and Their Cellular Impact on Colon LS 174T and Prostate DU 145 Cancer Cells
Source: Molecules. 2024 Nov 26;29(23):5582. doi: 10.3390/molecules29235582 (PMC11644013; doi:10.3390/molecules29235582)
Supplement: Supplementary file 1 [file molecules-29-05582-s001.zip › molecules-3272741-supplementary.pdf]

Unsymmetrical Bisacridines' Interactions with ABC Transporters and Their Cellular Impact on Colon LS 174T and Prostate DU 145 Cancer Cells

Monika Pawłowska, Jolanta Kulesza, Ewa Paluszkiewicz, Ewa Augustin* and Zofia Mazerska *

Department of Pharmaceutical Technology and Biochemistry, Faculty of Chemistry, Gdańsk University of Technology, Gabriela Narutowicza Str. 11/12, 80-233 Gdańsk, Poland; monika.pawlowska@pg.edu.pl (M.P.); jolanta.kulesza@pg.edu.pl (J.K.); ewa.paluszkiewicz@pg.edu.pl (E.P.)
* Correspondence: ewa.augustin@pg.edu.pl (E.A.); zofia.mazerska@pg.edu.pl (Z.M.); Tel.: +48-58-347-14-68 (E.A.); +48-58-347-12-97 (Z.M.)

Supplementary Materials

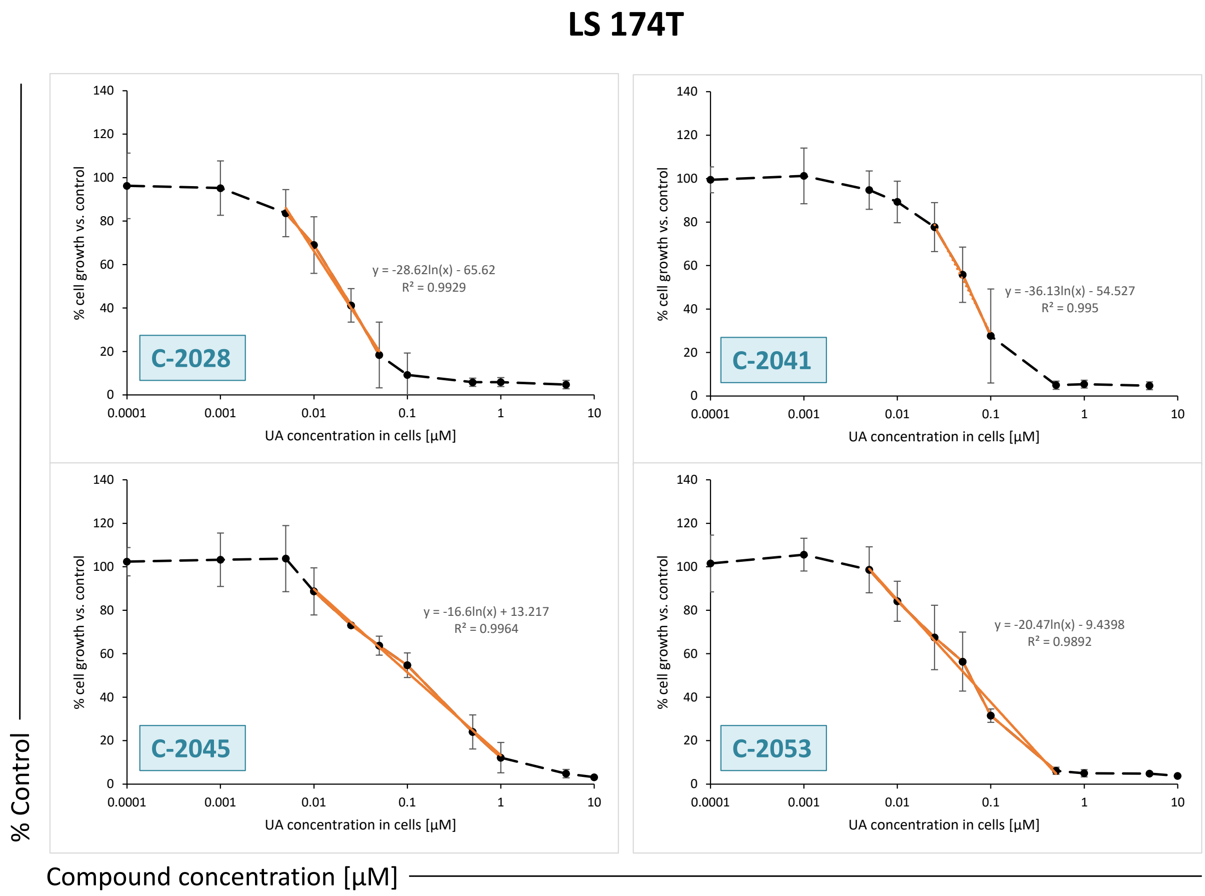

Figure S1. Growth inhibition curves of LS 174T cells after exposure to UAs compounds. Cells were incubated with increasing concentrations of C-2028, C-2041, C-2045, and C-2053 for 72 h and cytotoxic activity was assessed using MTT assay (n≥6).

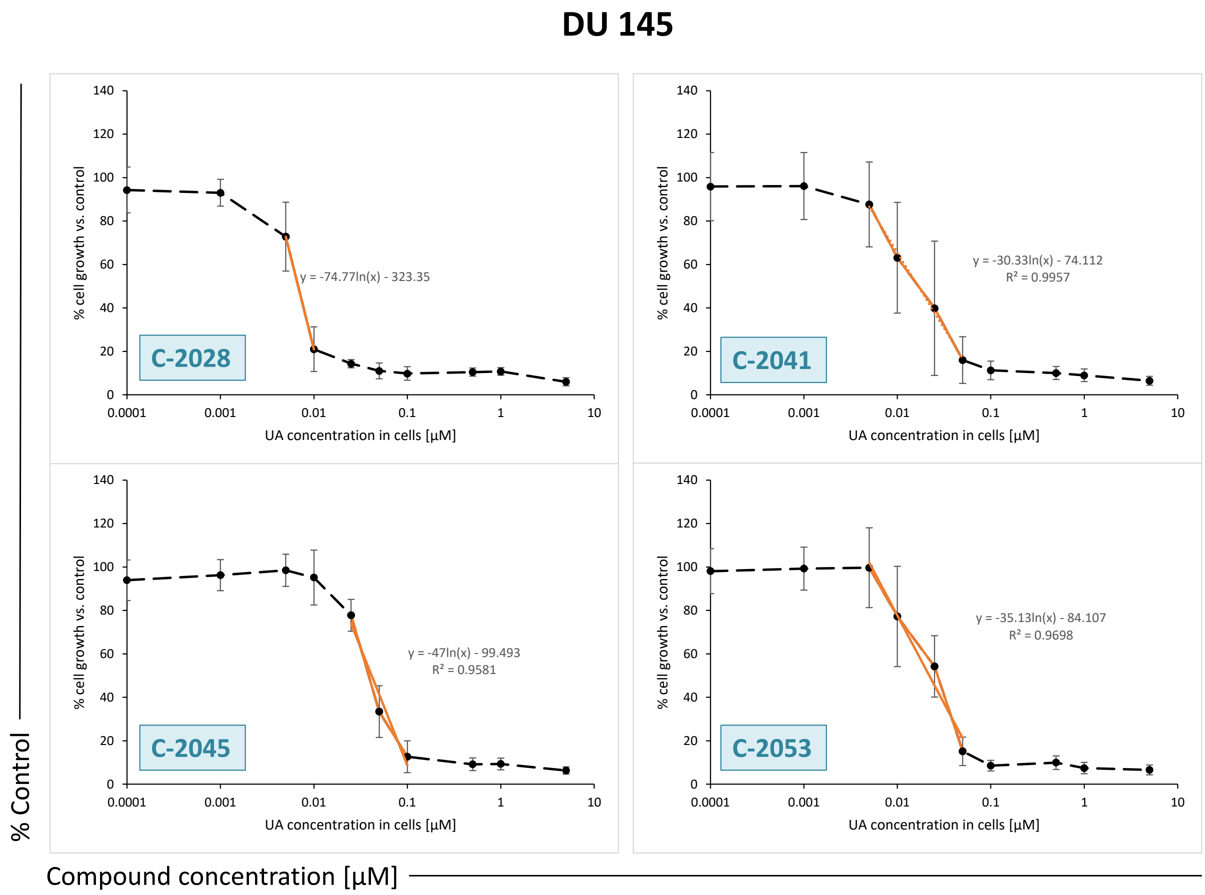

Figure S2. Growth inhibition curves of DU 145 cells after exposure to UAs compounds. Cells were incubated with increasing concentrations of C-2028, C-2041, C-2045, and C-2053 for 72 h and cytotoxic activity was assessed using MTT assay (n≥6).

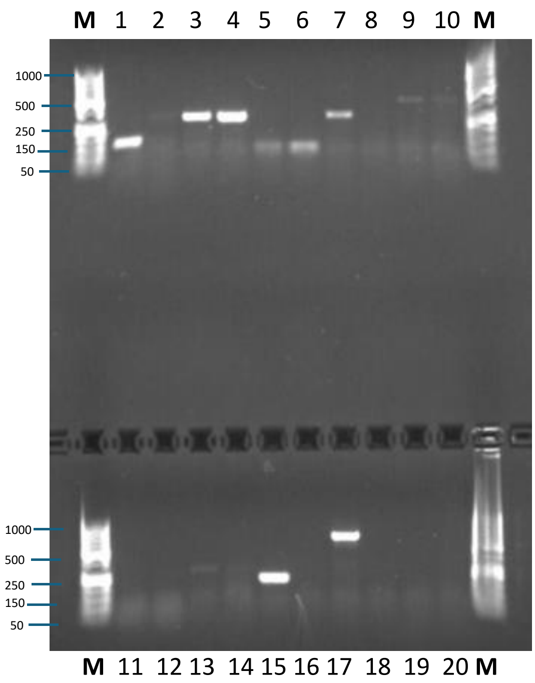

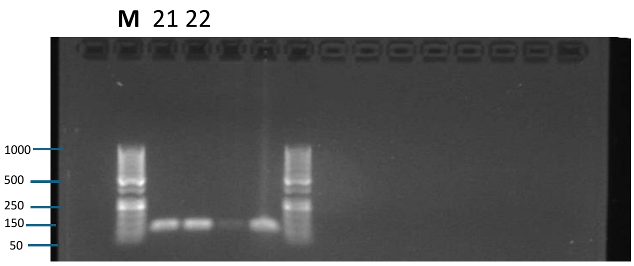

M – marker
1 – LS 174T *ABCB1*
2 – DU 145 *ABCB1*
3 - LS 174T *ABCC1*
4 - DU 145 *ABCC1*
5 - LS 174T *ABCC2*
6 – DU 145 *ABCC2*
7 – LS 174T *PXR*
8 – DU 145 *PXR*
9 – LS 174T *CYP1A2*
10 – DU 145 *CYP1A2*
11 – LS 174T *CYP2C9*
12 – DU 145 *CYP2C9*
13 – LS 174T *CYP3A4*
14 – DU 145 *CYP3A4*
15 – LS 174T *UGT1A1*
16 – DU 145 *UGT1A1*
17 – LS 174T *UGT1A10*
18 – DU 145 *UGT1A10*
19 – LS 174T *UGT2B7*
20 – DU 145 *UGT2B7*
21 – LS 174T *BACTIN*
22 – DU 145 *BACTIN*

Figure S3. Original photos taken for reverse transcript PCR analysis which are presented in Figure 2.

LS 174T cells

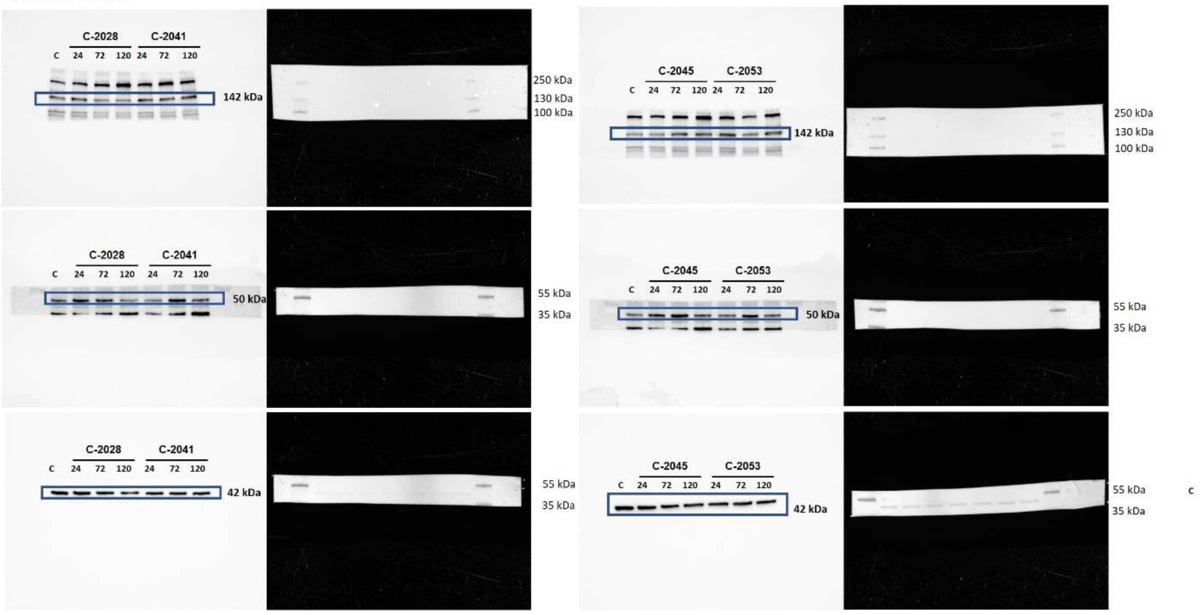

Figure S4. Original photos taken for Western blot analysis which are presented in Figure 5 taken for LS 174T cells.

DU 145 cells

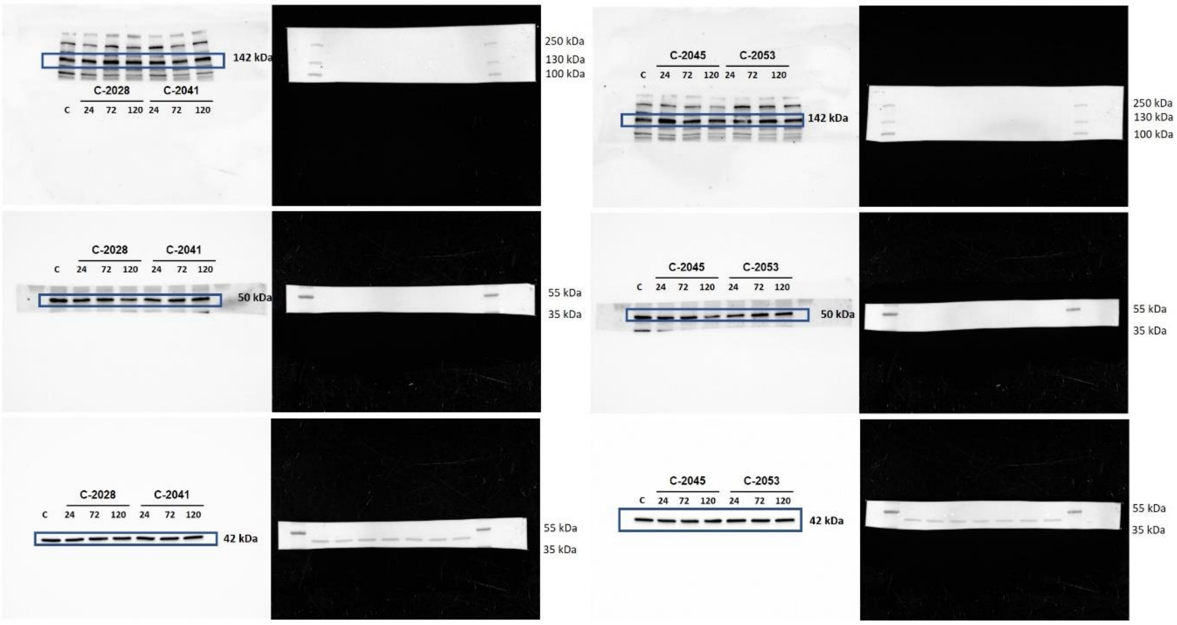

Figure S5. Original photos taken for Western blot analysis whioch are presented in Figure 5 taken for DU 145 cells.

Table S1. Cell cycle distribution of LS 174T cells after treatment with C-2028, C-2041, C-2045, and C-2053 (at IC_{80} dose); n≥3.

LS 174T						
**Compound**	**Incubation time [h]**	**Phase of cell cycle**				
		**Sub-G1**	**G1**	**S**	**G2/M**	**Poli**
**Control**	**24**	6.42 ± 2.23	55.70 ± 2.45	11.15 ± 0.83	11.35 ± 1.45	14.70 ± 4.38
**C-2028**	**24**	18.45 ± 6.49	39.35 ± 5.42	15.58 ± 1.52	14.65 ± 2.63	10.53 ± 1.87
	**72**	13.20 ± 3.91	51.23 ± 7.13	11.75 ± 1.10	12.00 ± 1.10	11.33 ± 2.93
	**120**	22.60 ± 6.97	45.40 ± 5.99	11.38 ± 1.39	11.38 ± 1.15	8.10 ± 0.30
**C-2041**	**24**	42.14 ± 13.55	27.46 ± 8.92	13.86 ± 2.14	7.28 ± 2.47	9.23 ± 4.05
	**72**	22.57 ± 3.33	40.53 ± 2.42	12.60 ± 0.82	10.73 ± 1.72	13.57 ± 2.00
	**120**	42.17 ± 17.83	34.30 ± 12.68	6.40 ± 0.72	8.77 ± 2.27	8.37 ± 2.26
**C-2045**	**24**	17.73 ± 5.00	33.60 ± 1.40	13.97 ± 0.91	15.27 ± 2.73	19.43 ± 3.07
	**72**	23.13 ± 11.61	36.25 ± 7.89	14.50 ± 1.68	12.25 ± 2.28	13.88 ± 1.75
	**120**	33.25 ± 9.26	32.90 ± 2.55	11.80 ± 0.14	12.25 ± 3.04	9.80 ± 3.54
**C-2053**	**24**	23.28 ± 9.07	30.98 ± 4.83	15.83 ± 2.15	11.28 ± 2.50	18.65 ± 3.82
	**72**	30.20 ± 4.05	29.93 ± 1.30	15.73 ± 1.36	10,57 ± 0.81	13,57 ± 2.11
	**120**	46.75 ± 0.07	19.90 ± 2.40	12.90 ± 1.41	12.00 ± 3.39	8.45 ± 0.49

Table S2. Cell cycle distribution of DU 145 cells after treatment with C-2028, C-2041, C-2045, and C-2053 (at IC_{80} dose); n≥3.

DU 145						
**Compound**	**Incubation time [h]**	**Phase of cell cycle**				
		**Sub-G1**	**G1**	**S**	**G2/M**	**Poli**
**Control**	**24**	4.00 ± 1.65	63.97 ± 5.71	11.50 ± 3.20	16.20 ± 5.17	4.33 ± 2.15
**C-2028**	**24**	5.03 ± 2.97	35.90 ± 2.12	38.53 ± 2.43	16.43 ± 0.78	4.10 ± 1.25
	**72**	26.33 ± 6.11	27.30 ± 4.72	28.77 ± 4.37	12.37 ± 7.14	5.23 ± 0.93
	**120**	55.63 ± 13.99	17.03 ± 8.66	17.57 ± 4.41	6.03 ± 1.55	3.73 ± 1.36
**C-2041**	**24**	9.8 ± 8.88	41.53 ± 3.77	34.00 ± 9.73	10.53 ± 0.78	4.13 ± 0.46
	**72**	26.87 ± 2.57	34.73 ± 2.64	25.20 ± 1.11	8.60 ± 4.84	4.60 ± 1.04
	**120**	43.93 ± 6.36	27.17 ± 2.71	20.07 ± 2.93	4.93 ± 1.38	3.90 ± 0.70
**C-2045**	**24**	6.33 ± 6.28	34.90 ± 4.91	40.47 ± 2.86	13.77 ± 2.73	4.53 ± 0.41
	**72**	26.10 ± 3.75	22.43 ± 2.65	36.47 ± 4.36	9.83 ± 3.90	5.17 ± 1.82
	**120**	54.55 ± 15.90	13.75 ± 4.26	20.58 ± 7.37	7.03 ± 3.63	4.10 ± 1.60
**C-2053**	**24**	1.80 ± 0.75	33.20 ± 5.48	45.07 ± 8.28	15.00 ± 3.86	4.93 ± 0.57
	**72**	22.13 ± 7.13	22.13 ± 1.50	37.03 ± 3.41	12.87 ± 8.53	5.83 ± 0.91
	**120**	51.33 ± 10.43	14.93 ± 4.41	22.43 ± 5.65	7.28 ± 3.01	4.05 ± 1.15

Table S3. Analysis of asymmetry and permeability of cell membrane in LS 174T cells after treatment with C-2028, C-2041, C-2045, and C-2053 (at IC_{80} dose); n≥4.

LS 174T					
**Compound**	**Incubation time [h]**	**A^-PI^-**	**A^+PI^-**	**A^+PI^+**	**A^-PI^+**
**Control**	**24**	83.18 ± 1.56	4.35 ± 1.18	7.00 ± 2.75	5.48 ± 2.05
**C-2028**	**24**	78.00 ± 4.82	4.20 ± 1.15	12.57 ± 4.85	5.27 ± 1.20
	**72**	74.90 ± 8.83	9.33 ± 4.47	8.25 ± 2.43	7.53 ± 2.93
	**120**	69.30 ± 6.74	18.00 ± 10.34	7.00 ± 1.81	5.68 ± 3.06
**C-2041**	**24**	69.80 ± 3.39	6.45 ± 0.07	18.15 ± 0.92	5.60 ± 2.40
	**72**	66.08 ± 15.11	17.15 ± 7.29	11.80 ± 6.68	5.00 ± 1.62
	**120**	48.00 ± 7.66	40.98 ± 6.81	7.70 ± 1.02	3.36 ± 1.13
**C-2045**	**24**	76.00 ± 6.75	5.40 ± 2.67	11.87 ± 4.61	7.00 ± 2.65
	**72**	73.62 ± 9.18	12.08 ± 7.05	8.06 ± 3.49	6.26 ± 2.67
	**120**	71.03 ± 4.13	18.87 ± 3.94	6.10 ± 0.92	4.03 ± 0.55
**C-2053**	**24**	75.58 ± 4.27	9.48 ± 2.13	10.70 ± 2.20	4.25 ± 0.98
	**72**	72.50 ± 9.37	13.00 ± 7.79	9.00 ± 1.51	5.50 ± 0.36
	**120**	65.10 ± 11.69	24.13 ± 11.43	6.40 ± 0.75	4.37 ± 0.32

Table S4 Analysis of asymmetry and permeability of cell membrane in DU 145 cells after treatment with C-2028, C-2041, C-2045, and C-2053 (at IC_{80} dose); n≥4.

DU 145					
**Compound**	**Incubation time [h]**	**A^-PI^-**	**A^+PI^-**	**A^+PI^+**	**A^-PI^+**
**Control**	**24**	95.83 ± 0.45	0.93 ± 0.35	2.33 ± 0.55	0.90 ± 0.78
**C-2028**	**24**	95.07 ± 0.78	1.67 ± 0.50	2.13 ± 0.45	1.10 ± 0.26
	**72**	85.53 ± 2.94	1.47 ± 0.74	10.33 ± 3.60	2.70 ± 0.95
	**120**	65.22 ± 3.59	10.26 ± 1.18	19.45 ± 2.31	3.30 ± 1.18
**C-2041**	**24**	93.93 ± 0.92	2.30 ± 0.46	2.50 ± 0.66	1.27 ± 0.32
	**72**	79.00 ± 3.82	2.53 ± 0.81	15.90 ± 5.11	2.53 ± 0.57
	**120**	59.32 ± 6.68	11.34 ± 1.30	22.68 ± 2.74	4.68 ± 1.59
**C-2045**	**24**	95.37 ± 0.76	1.30 ± 0.20	2.10 ± 0.53	1.23 ± 0.42
	**72**	78.10 ± 3.99	2.70 ± 0.75	16.43 ± 2.66	2.73 ± 0.93
	**120**	52.46 ± 3.07	13.74 ± 3.23	28.08 ± 3.69	3.74 ± 1.84
**C-2053**	**24**	95.10 ± 0.36	1.43 ± 0.12	2.23 ± 0.46	1.27 ± 0.32
	**72**	78.30 ± 0.66	2.73 ± 0.70	16.30 ± 0.61	2.63 ± 0.31
	**120**	60.66 ± 8.65	11.00 ± 3.87	24.22 ± 5.80	4.16 ± 1.30
